# Novel derivative of Paeonol, Paeononlsilatie sodium, alleviates behavioral damage and hippocampal dendritic injury in Alzheimer's disease concurrent with cofilin1/phosphorylated-cofilin1 and RAC1/CDC42 alterations in rats

**DOI:** 10.1371/journal.pone.0185102

**Published:** 2017-09-21

**Authors:** Fei Han, Ting-Ting Zhuang, Jing-Jing Chen, Xiu-Ling Zhu, Ya-Fei Cai, Ya-Ping Lu

**Affiliations:** 1 College of Life Science, Anhui Normal University, Wuhu, China; 2 Department of Anatomy, Wannan Medical College, Wuhu, China; Universita degli Studi di Torino, ITALY

## Abstract

Alzheimer’s disease (AD) is a typical hippocampal amnesia and the most common senile dementia. Many studies suggest that cognitive impairments are more closely correlated with synaptic loss than the burden of amyloid deposits in AD progression. To date, there is no effective treatment for this disease. Paeonol has been widely employed in traditional Chinese medicine. This compound improves learning behavior in an animal model; however, the mechanism remains unclear. In this study, Paeononlsilatie sodium (Pa), a derivative of Paeonol, attenuated *D*-galactose (*D*-gal) and AlCl_3_-induced behavioral damages in rats based on evaluations of the open field test (OFT), elevated plus maze test (EPMT), and Morris water maze test (MWMT). Pa increased the dendritic complexity and the density of dendritic spines. Correlation analysis indicated that morphological changes in neuronal dendrites are closely correlated with behavioral changes. Pa treatment reduced the production of Aβ, affected the phosphorylation and redistribution of cofilin1 and inhibited rod-like formation in hippocampal neurons. The induction of *D*-gal and AlCl_3_ promoted the expression of RAC1/CDC42 expression; however, the tendency of gene expression was inhibited by pretreatment with Pa. Taken together, our results suggest that Pa may represent a novel therapeutic agent for the improvement of cognitive and emotional behaviors and dendritic morphology in an AD animal model.

## Introduction

Alzheimer’s disease (AD) is the most common form of elderly dementia and has been generally regarded as an amnesic syndrome of hippocampal type, which represents the most important clinic feature for the diagnosis of typical AD [1, 2] and accounts for 50%-60% of all dementia cases [3]. In the Western world, the percentage of AD dementias is less than 1% in individuals aged 60–64 years, whereas it is approximately 24% to 33% in individuals aged 85 years or older [3]. Globally, more than 40 million individuals over 60 years have been estimated to have AD dementia, and the number of patients will double every 20 years until at least 2050 [4, 5].

The marked pathological features of AD include the formations of extracellular senile plaques from abnormally folded Aβs and intracellular neurofibrillary tangles from hyperphosphorylated tau proteins [6]. Aβ is considered the trigger in the disease process [7–10]; however, it is also a controversial marker [11]. The oligomers of Aβ, particularly soluble oligomers, such as “Aβ-Derived Diffusible Ligands” (ADDLs), which are mainly trimeric to 12meric Aβ_1-42_-oligomers, are considered to be the most cytotoxic forms [12–14]. These oligomers play an essential role during the occurrence and development of AD [13–15].

Dendritic spines undertake an important role in information processing in the brain, particularly for excitatory synaptic transmission [16–18] and plasticity [19]. In AD pathogenesis, synaptic loss contributes to cognitive impairments more than the burden of amyloid plaques [20, 21]. Previous studies in clinical settings [22] and animal models of AD [23–25] have confirmed the significant decrease in the number and morphological changes in dendritic spines in the neocortex and hippocampus compared with age-matched controls [17].

Aβ oligomers bind to various membrane receptors [26], such as glutamate receptors [27–29] and LilrB2 (murine PirB) [30], and transmit signals to GTPase-mediated different pathways to affect the activation/deactivation of cofilin1 and the formation of cofilin-actin rods, which ultimately affects the synaptic transmission and survival of postsynaptic neurons [17, 19].

To date, there is no effective treatment for this disease [31]. Paeonol (2’-hydroxy-4’-methoxyacetophenone, C_9_H_10_O_3_) is a phenolic acid compound extracted from the famous Cortex Moutan, which has been widely employed in traditional Chinese medicine (TCM). It is well established that Paeonol exerts anti-inflammatory activities to relieve ovalbumin-induced asthma [32] and cigarette smoke-induced lung inflammation [33] in a murine model, free-radical scavenging properties to prevent against neurotoxicity in vitro [34, 35], and anti-tumor effects in culture cells [36, 37]. Paeonol administration may reduce ischemia-reperfusion injured cerebral infarction [38] and ameliorate cognitive deficits in streptozotocin-induced diabetic rats [39].

Recent studies have indicated that Paeonol attenuates oxidative stress to protect against acetaminophen-induced hepatotoxicity in mice [40], reduces neuronal apoptosis in myocardial infarcted rats by inhibiting oxidative stress through the Nrf2-HO-1 and PI3K-Akt pathways [41], and alleviates cerebral ischemic injuries in mice by upregulating the expressions of pAkt, Nrf2, and HO-1 and ameliorating BBB permeability [42]; it also attenuates lipopolysaccharide-induced depressive-like behaviors in mice [43]. Paeonol applications improve Alzheimer’s disease-like behaviors in a rat model [44]; however, the mechanism remains unclear. To reduce Aβ oligomers and develop a novel compound to treat AD, in this study, we used Paeononlsilatie sodium (a derivative of Paeonol) to treat an AD animal model and investigated its potential mechanisms.

## Experimental procedures

### Drugs and chemicals

Paeononlsilatie sodium injection (C_9_H_9_NaO_6_S, a derivative of Paeonol, 0.1 g/2 ml), which has the same pharmacological effects as Paeonol and has been approved by the Chinese FDA for clinical use in the treatment of muscle pain, arthralgia, rheumatism, neuralgia and abdominal pain (No. H20064790, http://drugs.medlive.cn/drugref/html/18322.shtml), was purchased from Jinling Pharmaceutical Company (Nanjing, China). *D*-gal was purchased from Sangon Biotech (Shanghai, China). AlCl_3_ (Al) was purchased from Guangzhou Chemical Reagent Factory (Guangzhou, China). CDC42 and RAC1 antibodies were purchased from Boster Biological Technology Co., LTD (Wuhan, China). Phospho-Tau (Ser202) and Aβ_1–42_ antibodies (used to recognize the Aβ fibrils) were purchased from Bioss Biological Technology Co., LTD (Beijing, China). RHOA antibody was purchased from Sangon Biological Technology Co., LTD (Wuhan, China). A11 antibody (used to recognize the Aβ oligomers [45], SAB5200113) was purchased from Sigma-Aldrich (St. Louis, USA). Cofilin1 antibody and DAPI were purchased from Santa Cruz Biotechnology (Shanghai, China). Other chemicals were purchased from Sigma.

### Ethical approval of the study

Adult male Sprague–Dawley (SD) rats (180–200 g) were obtained from Shandong Experimental Animal Center (license number: SCXK20140007; Jinan, China) and housed at 21 ± 2°C, with a light cycle between 08:00 h and 20:00 h. Food and water were provided *ad libitum*. Animals were treated according to the Guidelines of the Regulations of Experimental Animal Administration issued by the State Committee of Science and Technology of the People’s Republic of China on November 14, 1988. All animal experiments were conducted with the approval of the Animal Use and Care Committee of Anhui Normal University (2015002).

### Establishment of AD rat model

AlCl_3_ and *D*-gal co-induced animal models may be used to characterize AD-like behavioral and pathological features, as well as pathologic processes without the genetic background of gene mutations and have thus been widely used in AD-related studies [46–54]. In this study, *D*-gal was dissolved in physiologic saline (0.9%) and injected (100 mg/kg/day, once per day, s.c.). AlCl_3_ was dissolved in double-distilled water (DDW) and administered (40 mg/kg/day, once per day, i.g.). Model induction lasted for 42 days.

### Group and treatment

Following a one-week adaptation period, the rats were randomly divided into the control group (CG), *D*-gal + AlCl_3_ group (DA group treated with *D*-gal + AlCl_3_ for 42 days), Pa treatment group (50 mg/kg, i.p., 1 hour before treatment with AlCl_3_ + *D-*gal) for 2 weeks (Pa2 group treated with AlCl_3_ + *D-*gal + saline water (pretreatment) for 28 days, followed by AlCl_3_ + *D-*gal + Pa for 14 days), and Pa treatment group for 6 weeks (Pa6 group treated with AlCl_3_ + *D-*gal + Pa for 42 days). The induction of CG and the pretreatments for CG or DA were all volume-matched saline water.

### Behavioral tests

#### OFT

The OFT was used to evaluate animal locomotor activity and emotional response [55]. The apparatus was opaque and black all around, including a square 80 cm×80 cm surrounded by a height of 40 cm walls, and the bottom was divided into 25 quadrates of 16 cm×16 cm by a white line. In a quiet environment, each rat was placed in the center area, which was defined as 9 squares in the center, and the behaviors were recorded for 5 min. The device was cleaned with 70% ethanol thoroughly after each trial. The number of squares crossed, number of clean movements, and number of rears were recorded. The animals were placed in the laboratory room 30 min prior to testing to adapt to the environment. The observers were blind to the rats of the different treatment groups.

#### EPMT

The EPMT has typically been used to evaluate anxiety behaviors [56]. The apparatus consisted of four black arms (50 cm×10 cm) and a central square platform (10 cm×10 cm). Two of the arms were closed arms with walls (40 cm height), and the other two arms were open without walls. The maze was elevated 50 cm from the floor. The rats were placed onto the center platform of the maze facing an open arm, and their activities were recorded for 5 minutes. When a rat placed its four paws into an arm, it was counted as entry in the arm. The maze was cleaned with 70% ethanol thoroughly after each trial. The following parameters were recorded: the time spent in the open arms, the time spent in the closed arms, and the number of head dipping. The experimenters who recorded the data were unaware of the grouping.

#### MWMT

Spatial learning and memory capability were assessed using a modified MWMT. The pool (1.5 m in diameter, 50 cm in height) was filled with water to a depth of 30 cm. A circular escape platform (15 cm in diameter) was submerged 2 cm below the water surface. The water was clouded with black food coloring to prevent the visual image of the submerged platform. On the last five days during treatment for each group, the rats were trained to find a concealed platform in the MWM for five consecutive days. The MWM test was conducted according to previously described methods [57]. Briefly, the acquisition phase comprised four trials per day in which the animals learned to find the concealed platform. The rats were placed facing the wall at one of four designated start points in each trial. Each rat was allowed to find the platform for 60 s and remained on the platform for 20 s. If the rat could not reach the platform within 60 s, it was gently guided to the platform and remained for 20 s, and the results (time taken to find the platform) were recorded as 60 s. The average escape latencies of the four trials per day were used for statistical analysis. A longer time spent in finding the platform was used to assess the extent of learning impairment.

Twenty-four hours after the final acquisition trial, a single 60 s probe task was tested following the removal of the platform from the pool. The probe trials were recorded with a video camera to calculate the time the rats spent in the target quadrant. The longer the rat remained in the target quadrant, the better it scored for spatial memory. The time was manually recorded with a stopwatch (precision 1/100 seconds) by two observers, who were blind to the rats of the different treatment groups.

### Tissue preparation

Following the behavioral tests, the rats were deeply anesthetized with 1% carbrital and perfused (via a transcardial approach) with 0.9% saline followed by 1) euthanasia via decapitation and isolation of hippocampal tissue from the brain, half of which was stored in Golgi-cox solution, while the other half of the hippocampus was stored in a -80°C refrigerator for further Western blot analysis; 2) perfusion with 4% paraformaldehyde and euthanasia, followed by the hippocampal tissues being collected and embedded in paraffin wax. The paraffin-embedded tissues were cut with a Leica microtome (RM2235) into serial coronal sections (6 μm).

### Golgi-Cox staining

The Golgi–Cox technique was mainly conducted as described in the literature [58]. In brief, brain tissues were stored in Golgi-Cox solution in the dark at 37°C for 48 h and subsequently sectioned (200 μm thick coronal sections) using a vibratome (NVSL/NVSLM1). The sections were then immersed in alcohol (50%) for 5 min, ammonium hydroxide for 5–10 min, and 5% sodium thiosulfate in the dark for 10 min and serially dehydrated in solute alcohol, including 70%, 80%, and 95% alcohol (7 min × 2 for each) and 99% 1-butanol for 7 min; the samples were subsequently cleaned in xylene for 5 min and medium mounted with coverslips using Rhamsan gum.

### Immunohistochemistry and immunofluorescence

After being deparaffinized and rehydrated, the sections were treated with 0.3% hydrogen peroxide in phosphate buffered solution (PBS, pH 7.0) that contained 0.3% Triton X-100 for 30 min. The sections were subsequently treated twice (2×10 min) by microwaves (700 W) in 0.05 M citrate buffered saline (pH 6.0). After being washed in PBS, the sections were blocked with normal bovine serum in PBS for 1 h at 37°C, followed by incubation with primary antibodies [rabbit Aβ_1–42_ pA (1:200), rabbit phospho-tau (p-tau) pA (1:200) or mouse cofilin1 mA (1:200)] overnight at 4°C. Single labeling with DAB (Aβ_1–42_ and p-tau immunoreactivity): after being washed, the sections were incubated with the secondary antibody goat anti-rabbit (Boster Biotech, Wuhan, China) for 1 h at 37°C, which was performed as previously described [59]. Double labeling with fluorescein labeling methods previously described by our group [60]: after being washed, the sections were incubated with biotinylated horse anti-mouse IgG diluted in PBS (1:200) that contained 5% normal horse serum for 1 h and then incubated with Cy^TM^3-labeled streptavidin (Kirkegaard and Perry Laboratories) diluted in PBS (1:500) for 30 min at room temperature, followed by incubation with 0.05 M glycine-HCl buffer saline (pH 2.2) for 2 h at room temperature to quench additional antibodies; the samples were subsequently incubated with second primary antibodies against rabbit p-cofilin1 pA (1:200) for 24 h at 4°C and incubated with the fluorescein isothiocyanate (FITC)-conjugated goat anti-rabbit IgG (Vector Laboratories, Burlingame, CA) for 1 h, diluted in PBS (1:100). Finally, the sections were coverslipped with glycerin. Control samples were simultaneously performed following the same procedures as the test samples with the exception that the primary antibodies were omitted.

### Quantification of dendrites and spines

Quantitative methods of the dendritic branch and length have been previously described in the literatures [61, 62]. Briefly, the regions tested were determined at a low magnification according to Paxinos et al [63], and > 5 neurons in each hippocampal region per rat were selected to acquire their photographs, which were used for quantificational analysis. The criteria used to select neurons for quantitative analysis have been previously described [61, 64, 65]. Each selected neuron was analyzed using ImageJ software. Neuronal branches were traced by the Neuron J plug-in to count their total dendritic length, and dendritic intersections that cross the concentric circles were counted using the Sholl analysis plug-in protocol [65–67].

The analysis of the density and classification of dendritic spines were mainly based on the methods described in the previous literatures [61, 62]. Concisely, three independent coronal sections at the level of the lateral geniculate body per rat were used for analysis. At the apical proximal dendrites (< 50 μm from the center of the neuronal body), apical distal dendrites (> 150 μm from the center of neuronal body), and basal dendrites in CA1 and CA3, secondary or tertiary dendritic segments of pyramidal neurons were selected for analysis. In the DG, the apical dendritic segments at the outer 1/3 and 2/3 from the body of the granule neurons were selected for analysis. Z-stacks of Golgi-stained dendrites (up to 80 microns total on Z-axis; optical section thickness = 0.5 mm, i.e., 160 images per stack) were obtained at 60×6 magnification on an OLYMPUS FV1000. The Golgi spine images of the Z-stacks were analyzed using RECONSTRUCT software, which is freely available from http://synapses.clm.utexas.edu. The analysis was divided into three steps: step 1, series import and calibration; step 2, dendritic segment identification and measurement; and step 3, spine measurement and classification (S1 Fig).

### Image analysis and quantification

Image analysis and quantification of the histological sections were performed by one author who was not aware of the experimental protocol. Images were captured under a microscope (BX51; Olympus, Tokyo, Japan) equipped with a DP70 digital camera or Laser scanning confocal microscope (OLYMPUS FV1000) and analyzed using image analysis software (ImageJ). In serial sections between Bregma -2.52 and -4.80 mm, one section was selected at intervals of every sixth section. The percentage of hippocampal regions covered by Aβ immunopositive regions was measured to estimate the Aβ burden [68]. The F-actin density was assessed by the percentage of phalloidin positive staining in different regions of the hippocampus.

### Western blot analysis

For western immunoblot analysis, 10% (w/v) hippocampal homogenates were centrifuged at 12,000×g for 30 min at 4°C, and the supernatants were transferred to clean tubes and stored as previously described [69]. The total protein concentration in the supernatants was determined using the Bicinchoninic acid assay (BCA; Pierce). Samples (40 μg of total protein), mixed with an equal volume of Tricine sample buffer, were electrophoresed on various concentrations of Tris-tricine polyacrylamide gels (under nonreducing conditions) and transferred to nitrocellulose membranes. The blots were blocked with 5% nonfat dry milk in Tris-buffered saline Tween 20 (TBS-T) for 2 h at room temperature. RHOA (1:400), RAC1 (1:200), CDC42 (1:200), cofilin (1:100), p-cofilin (1:800), or A11 (1:800) antibodies were diluted in 0.1% BSA/ TBS-T, covered with plastic wrap and incubated for 12 h at 4°C. Bound antibodies were visualized with horseradish peroxidase-conjugated secondary antibodies and the ECL detection system (BEYOTIME Biological Technology Co., LTD, China). Densitometric analysis of antibody specific bands was performed with NIH ImageJ version 1.34 software.

### Statistical analyses

Analysis of variance (ANOVA) was used for comparisons of more than three groups, and a two-tailed *t* test was used for comparisons between two groups. The data of the spatial navigation tasks and the Sholl analysis were analyzed by a repeated ANOVA followed by post hoc Bonferroni’s multiple comparisons. Other data were analyzed via one-way ANOVA followed by *post hoc* Least-significant difference (LSD). All data were analyzed with SPSS v21.0 software (IBM, New York, NY, USA) and expressed as the mean ± standard error of the mean (SEM). *P* < 0.05 was considered to be statistically significant.

## Results

### Pa ameliorated *D*-gal and AlCl_3_-induced behavioral damage

**Fig 1:**
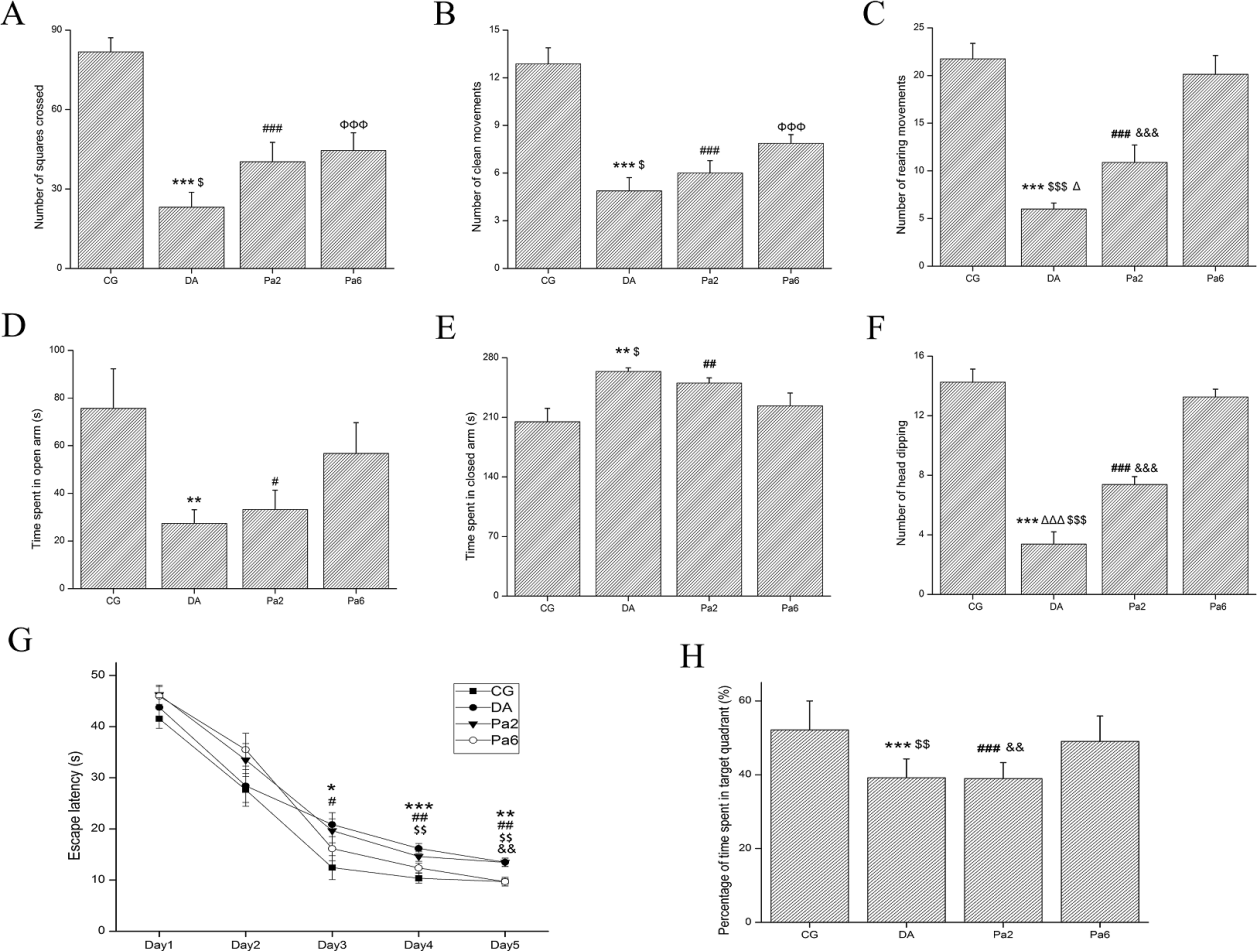
**Effects of Pa on the behavioral performances in the OFT (A-C), EPMT (D-F) and MWMT (G, H)** (n = 8–10). Pa treatment (50 mg/kg, i.p.) attenuated the *D*-gal and AlCl_3_-induced decrease in the number of squares crossed (**A**), the number of clean movements (**B**), and the number of rears (**C**) in the OFT. In the EPMT, *D*-gal and AlCl_3_ induced a decrease in the time spent in open arms (**D**), which was not significantly improved by Pa treatment. However, Pa attenuated the decrease in the number of head dipping (**F**) and alleviated the increase in the time spent in closed arms (**E**). (**G**) The average escape latencies of four acquisition trials per day in DA rats from days 3 to 5 were significantly more than those in CG rats. Pa pretreatment for 6 weeks produced significant protective effects, particularly at days 4~5. (**H**) Probe trials 24 h after acquisition trials indicated that animals in Pa6 pre-treated with Pa for 6 weeks spent more time in the target quadrant than animals in DA. Data expressed as the means ± SEM. *P<0.05, **P<0.01, ***P<0.001, DA versus CG; ^#^*P<*0.05, ^##^*P<*0.01, ^###^*P<*0.001, Pa2 versus CG; ^ΦΦΦ^*P<*0.001, Pa6 versus CG; ^Δ^*P<*0.05, ^ΔΔΔ^*P<*0.001, DA versus Pa2; ^$^*P<*0.05, ^$$^*P<*0.01, ^$$$^*P<*0.01, DA versus Pa6; ^&&^*P<*0.01, ^&&&^*P<*0.001, Pa2 versus Pa6.

To determine the preventive effects of Pa on *D*-gal and AlCl_3_-induced behavioral damage, rats were pre-treated with Pa (50 mg/kg) for 6 weeks. The OFT, EPMT and MWMT were employed to evaluate the animal behavioral performances. The OFT used in this study was represented by three parameters, including the number of squares crossed, the number of clean movements, and the number of rears. One-way ANOVA identified significant differences between the groups for these three parameters (*P<*0.001 for all). Compared with the CG, the three parameters all showed significant decreases observed in DA (*P<*0.001 for all). Pre-treatment with Pa significantly increased the number of squares (*P<*0.05), the number of clean movements (*P<*0.05), and the sum of rears (*P<*0.001) in Pa6 compared with DA (**Fig 1A–1C**).

As shown in **Fig 1D–1F**, there were significant effects of group on the time spent in the open arms (*P<*0.05), the time spent in the closed arms (*P<*0.01) and the number of head dipping (*P<*0.001) in the EPMT. Compared with DA, the rats in Pa6 showed a significant decrease in the time spent in the closed arms (*P<*0.05) and a significant increase in the number of head dipping (*P<*0.001).

In the MWMT, the average escape latencies of the four trials per day of each group are detailed in **Fig 1G**. A repeated ANOVA indicated significant effects of both factors (days: *F*_3,36_ = 795.499, *P*<0.001; group: *F*_3,36_ = 4.239, *P*<0.05) and no significant interaction (*F*_3,36_ = 2.584, *P>*0.05). On the fifth day, pairwise comparisons showed that significant differences were identified between CG and DA (*P<*0.01), as well as DA and Pa6 (*P<*0.01), in contrast to CG and Pa6 (*P>*0.05) (**Fig 1G**). The probe trial performances were assessed by removing the platform 24 h after the acquisition trials. Multiple comparisons indicated that the time spent in the target quadrant during the probe trial of the animals was significantly more in Pa6 than DA (*P<*0.01) (**Fig 1H**).

To evaluate the curative effects of Pa on *D*-gal and AlCl_3_-induced behavioral damage, rats were treated with Pa (50 mg/kg) for 2 weeks following administration with *D*-gal and AlCl_3_ for 4 weeks. Treatment with Pa for 2 weeks also increased the sum of rears in the OFT (*P<*0.05) (**Fig 1C**) and upregulated the number of head dipping in the EPMT (*P<*0.001) (**Fig 1F**). Our data suggest that Pa can alleviate *D*-gal and AlCl_3_-induced decreases in animal locomotor activity, performances of anxiety behaviors, and learning and memory impairments.

### Pa alleviated *D*-gal and AlCl_3_-induced Aβ and p-tau burdens in the hippocampus

**Fig 2:**
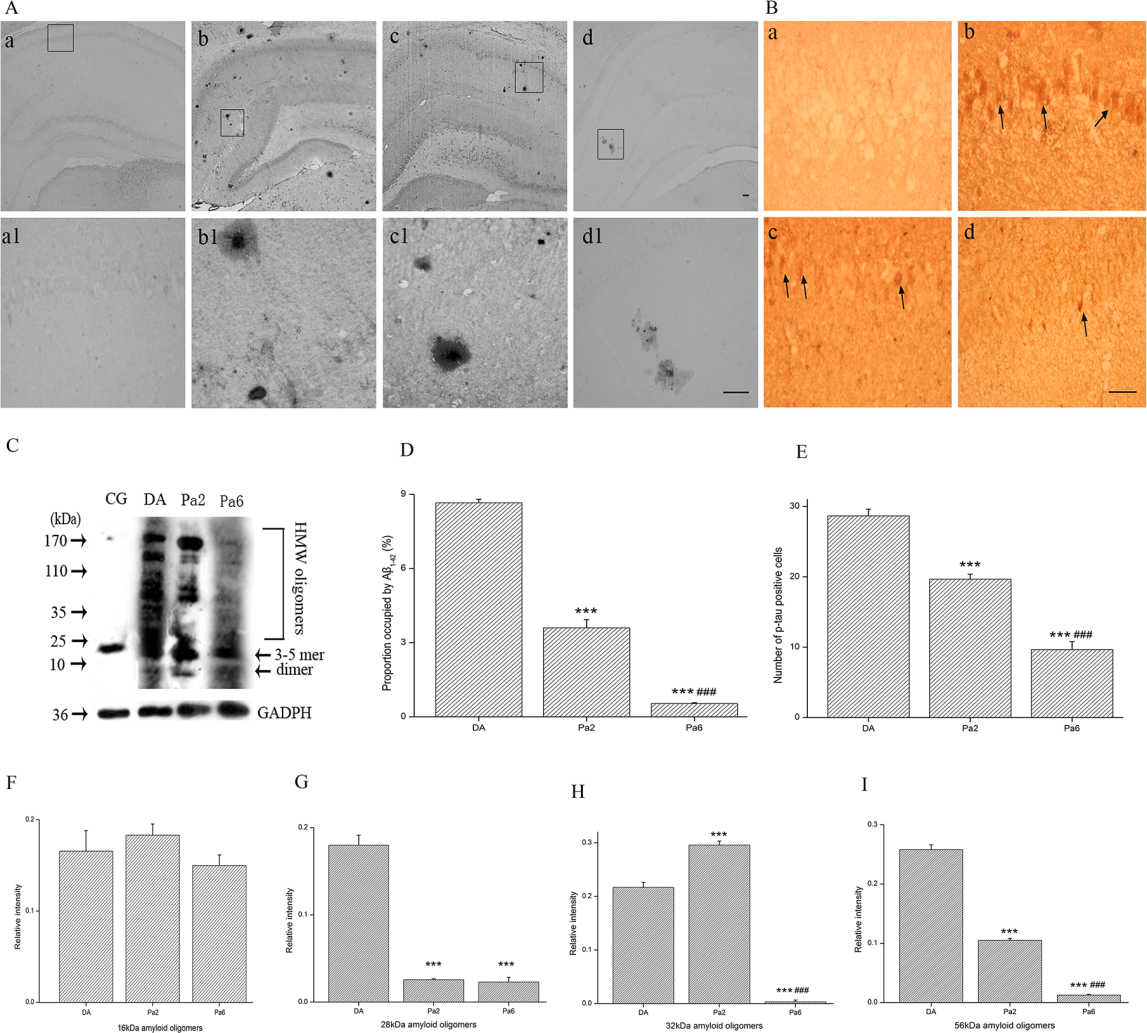
Pa alleviated *D*-gal and AlCl_3_-induced Aβ and tau burdens in the hippocampus. (**A**) Grayscale microscope pictures in immunohistochemical staining with Aβ_42_ antibody in the hippocampus in CG (a), DA (b), Pa2 (c), and Pa6 (d) and their magnifications (a’, b’, c’, and d’, respectively). (**B**) Phospho-tau immunohistochmical staining (p-Ser 202) in the hippocampus in CG (a), DA (b), Pa2 (c), and Pa6 (d). (**C**) Western blot bands with A11 antibody identifying different molecular weights of Aβ oligomers in the hippocampus in CG (left column), DA (middle left column), Pa2 (middle right column), and Pa6 (right column). Administration of Pa (50 mg/kg, i.p.) for 2 or 6 weeks significantly reduced the percentage area of Aβ_42_-positive deposits (**D**) and the number of p-tau positive cells (**E**). Pa did not affect the expression of 16 KD Aβ oligomers (**F**); however, it decreased the expression of 28 KD Aβ oligomers after the treatment of Pa for 2 or 6 weeks (**G**), downregulated the expression of 36 KD Aβ oligomers after the treatment of Pa for 6 weeks (**H**), and reduced the expression of 56 KD Aβ oligomers after the treatment of Pa for 2 or 6 weeks (**I**). Data expressed as the means ± SEM (n = 4~5). ***P<0.001, versus DA; ^###^*P<*0.001, versus Pa2. Scales: 100 μm in **A** (d1) and 50 μm in **B** (d).

The remarkable pathological features of AD include excess Aβ deposits and the formation of hyperphosphorylated-tau. Histological observation indicated that the Pa-pretreated rats had less Aβ_1-42_-positive deposits and p-tau positive inclusions than the vehicle-matched controls as visualized by immunostaining (using anti-Aβ_1–42_ and -p-tau antibodies) (**Fig 2A and 2B**). One-way ANOVA indicated differences between the groups in the percentage areas of the Aβ_1-42_-positive deposits (*P<*0.001) and the number of p-tau positive cells (*P<*0.001) (**Fig 2D and 2E**). Two-tailed *t* tests indicated that Pa treatment for two weeks and Pa pretreatment for six weeks led to 58.4% (*P<*0.001) and 93.8% (*P<*0.001) reductions of hippocampal Aβ_1–42_ burdens, respectively, and 35.4% (*P<*0.001) and 66.3% (*P<*0.001) reductions of hyperphosphorylated-tau burdens, respectively.

Western blot bands of the Aβ oligomers indicated visible differences between the groups (**Fig 2C**). What type of Aβ oligomers has neurotoxicity? Many studies have focused on ~56 KD [70], as well as ~32 KD Aβ oligomers [71–73]. In this study, we analyzed ~16 KD, ~28 KD, ~32 KD and ~56 KD Aβ oligomers. Pre-treatment with Pa for 6 weeks remarkably reduced the formations of Aβ oligomers of ~28 KD, ~32 KD and ~56 KD. The administration of Pa for 2 weeks also resulted in a significant reduction of Aβ oligomers of ~28 and ~56 KD (**Fig 2F–2I**). Our results indicate that Pa reduces Aβ deposits, hyperphosphorylated-tau formations, and toxic Aβ oligomer productions.

### Pa reduced dendritic spine loss and dendritic atrophy in the hippocampus

In general, it is accepted that toxic Aβ oligomers cause synaptic and dendritic spine loss [74–77], which are highly associated with the behavior abnormalities widely observed in several psychiatric disorders and neurodegenerative diseases, such as AD [16, 18, 19]. More than 90% of excitatory synapses are formed on dendritic spines that protrude from the main dendritic shaft (reviewed in Nimchinsky et al. 2002) [78]. Therefore, we evaluated the effects of Pa on *D*-gal and AlCl_3_-induced dendritic injury, including the dendritic branch and length and the density and types of dendritic spines, in hippocampal pyramidal and granular cells.

**Fig 3:**
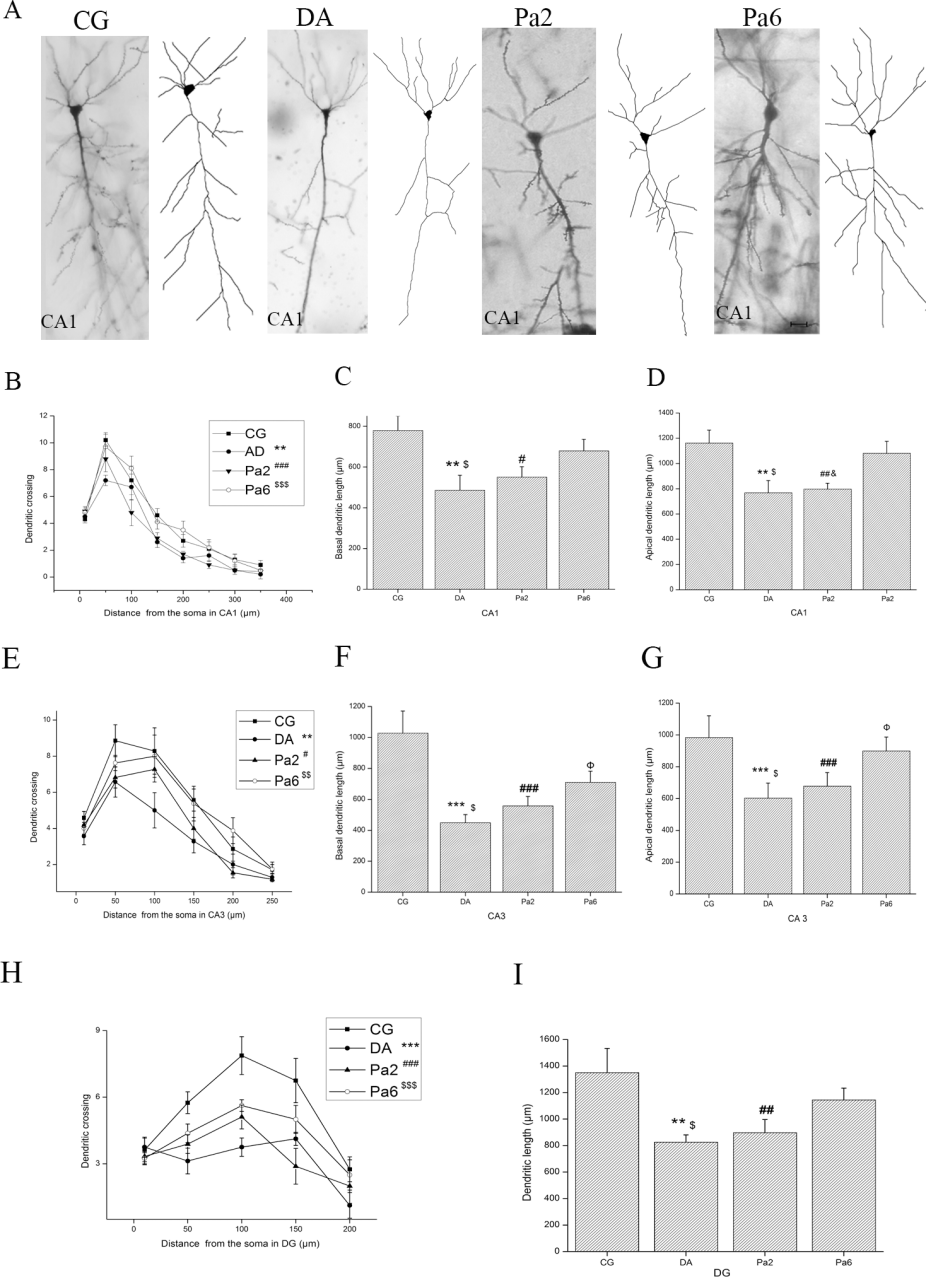
**Pa attenuated the decrease in dendritic length and branches in hippocampal CA1 (B-D), CA3 (E-G) and DG (H, I).** (**A**) CA1 pyramidal neurons and their traces drawn by ImageJ software. Pa pretreatment (50 mg/kg, i.p.) for 6 weeks significantly increased the number of dendritic branches in CA1 (**B**), CA3 (**E**), and DG (**H**), extended the length of basal dendrites in CA1 (**C**) and CA3 (**F**), and increased the length of apical dendrites in CA1 (**D**), CA3 (**G**), and DG (**I**). However, Pa treatment for 2 weeks did not significantly affect the dendritic branch or length in hippocampal neurons. Data expressed as the means ± SEM (n = 8~10). **P <0.01, ***P<0.001, DA versus CG; ^#^*P<*0.05, ^##^*P<*0.01, ^###^*P<*0.001, Pa2 versus CG; ^Φ^*P*<0.05, Pa6 versus CG; ^$^*P*<0.05, ^$$^*P*<0.01, ^$$$^*P*<0.001, DA versus Pa6; ^&^*P*<0.05, Pa2 versus Pa6.

**Fig 3A** presents the pyramidal neurons of CA1 in CG, DA, Pa2, and Pa6. Sholl analysis indicated that Pa pretreatment for 6 weeks prominently alleviated the *D*-gal and AlCl_3_-induced reduction of dendritic length and branching in both pyramidal cells of the CA1 (**Fig 3B–3D**) and CA3 (**Fig 3E–3G**) and granular cells of the DG (**Fig 3H and 3I**). Treatment with Pa for 2 weeks did not significantly affect the length of dendrites or the number of intersections.

**Fig 4:**
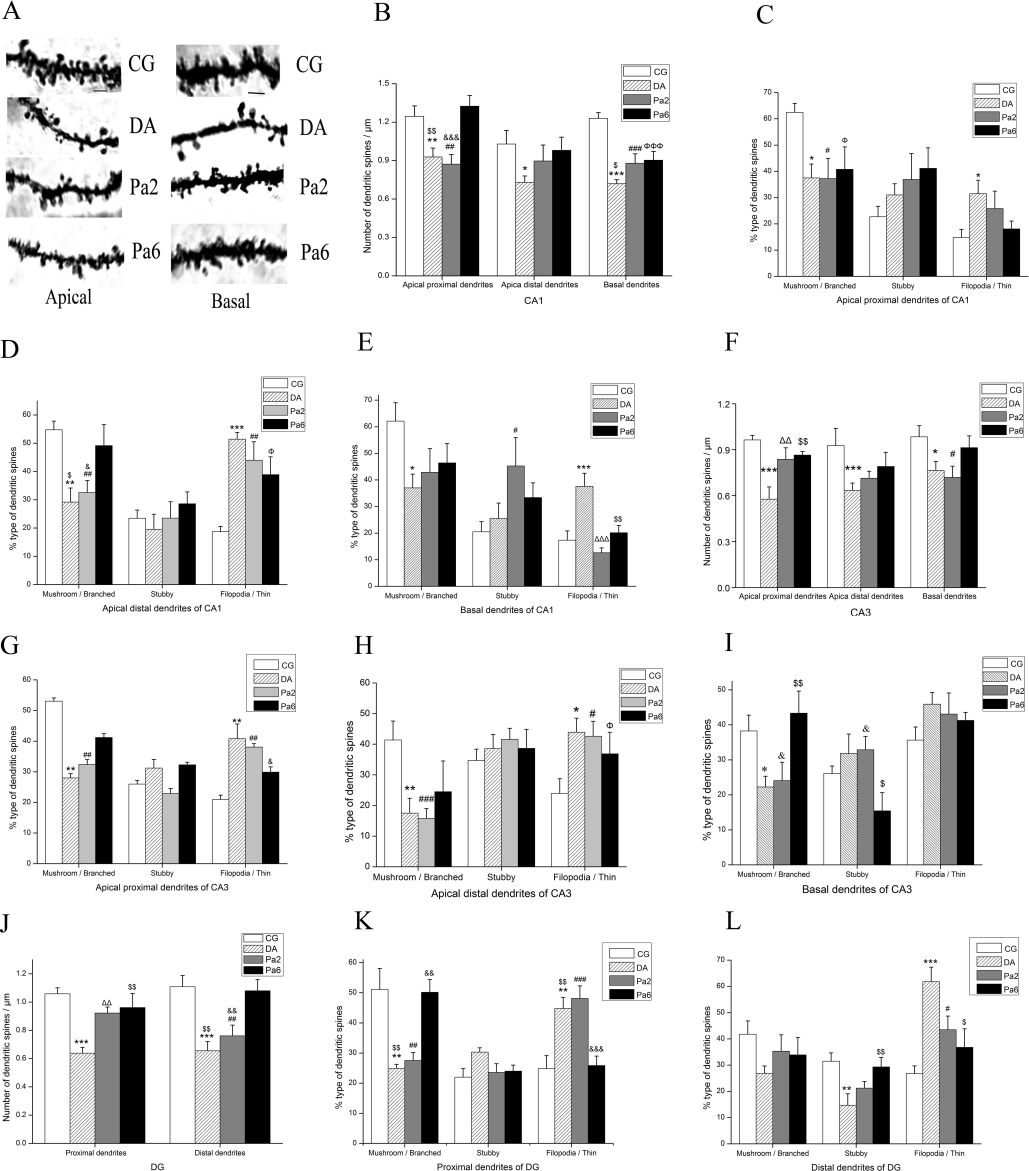
**Effects of Pa on density and type of dendritic spines in hippocampal CA1 (B-E), CA3 (F-I) and DG (J-L).** (**A**) Slice view acquired by Laser scanning confocal microscope (FV1000, 60×6 for objective magnification) at the apical proximal (left column) and basal (right column) dendritic segments stained by Golgi-Cox method in CA1 of CG, DA, Pa2, and Pa6. In CA1, Pa pretreatment for 6 weeks significantly increased the dendritic spine density in apical proximal dendrites and basal dendrites but not in apical distal dendrites (**B**), reduced the percentage of filopodia / thin in basal dendrites (**C**), and increased the percentage of mushroom / branched in apical distal dendrites (**E**); however, it did not significantly affect the percentage of various types of dendritic spines in apical proximal dendrites (**D**). Treatment with Pa for 2 weeks only reduced the percentage of filopodia / thin in basal dendrites. In CA3, pretreatment of Pa for 6 weeks increased the dendritic spine density in apical proximal dendrites (**F**), reduced the percentage of filopodia / thin in apical proximal dendrites (**G**), upregulated the percentage of mushroom / branched in basal dendrites (**I**), and downregulated the percentage of stubby in basal dendrites (**I**); however, it did not significantly affect the percentage of various types of dendritic spines in apical distal dendrites (**H**). Treatment of Pa for 2 weeks also increased the dendritic spine density in apical proximal dendrites (**F**). In the DG, the dendritic spine density of apical proximal and distal dendrites was higher in Pa-treated groups than vehicle-matched controls (**J**). The percentage of mushroom / branched was increased and the percentage of filopodia / thin was reduced in apical proximal dendrites after 6 weeks pretreatment with Pa (**K**), whereas the percentage of stubby was increased and the percentage of filopodia / thin was reduced in apical distal dendrites after 6 weeks pretreatment of Pa (**L**). Data expressed as the means ± SEM (n = 8~10). *P<0.05, **P<0.01, ***P<0.001, DA versus CG; ^#^*P<*0.05, ^##^*P<*0.01, ^###^*P<*0.001, Pa2 versus CG; ^Φ^*P<*0.05, ^ΦΦΦ^*P<*0.001, Pa6 versus CG; ^ΔΔ^*P<*0.01, ^ΔΔΔ^*P<*0.001, DA versus Pa2; ^$^*P<*0.05, ^$$^*P<*0.01, DA versus Pa6; ^&^*P<*0.05, ^&&^*P<*0.01, ^&&&^*P<*0.001, Pa2 versus Pa6.

As shown in **Fig 4A**, the slice view of dendritic segments indicated visible differences in the morphology and number of dendritic spines between the groups. In this study, three types of dendritic spines, mushroom / branched (MB, width > 0.6 μm or “branch”), stubby (ST, length: width ratio (LWR) ≤ 1 and length < 1 μm), and filopodia / thin (FT, length > 1 μm or LWR > 1), were automatically divided by reconstruct software by analyzing the Z-stacks. Pretreatment of Pa for 6 weeks significantly alleviated the *D*-gal and AlCl_3_-induced decrease in the spine density of the CA1 apical proximal dendrites and basal dendrites (**Fig 4B**), CA3 apical proximal dendrites (**Fig 4F**), and DG apical proximal and distal dendrites (**Fig 4J**). Pa pretreatment for 6 weeks substantially increased the percentage of MB spines in the CA1 apical distal dendrites (**Fig 4D**), CA3 basal dendrites (**Fig 4I**), and DG apical proximal dendrites (**Fig 4K**), upregulated the percentage of ST spines in the DG apical distal dendrites (**Fig 4L**), and reduced the percentage of FT spines in the CA1 basal dendrites (**Fig 4E**), as well as in the DG apical proximal (**Fig 4K**) and distal dendrites (**Fig 4L**). Treatment with Pa for 2 weeks also significantly alleviated the *D*-gal and AlCl_3_-induced decrease in the spine density of the CA3 apical proximal dendrites (**Fig 4F**) and DG apical proximal dendrites (**Fig 4J**). These data suggest that Pa reduces *D*-gal and AlCl_3_-induced dendritic spine loss, increases the percentage of MB spines, and downregulates the percentage of FT spines in a region-dependent manner.

### Pa alleviated *D*-gal and AlCl_3_-induced abnormalities of actin remodeling

**Fig 5:**
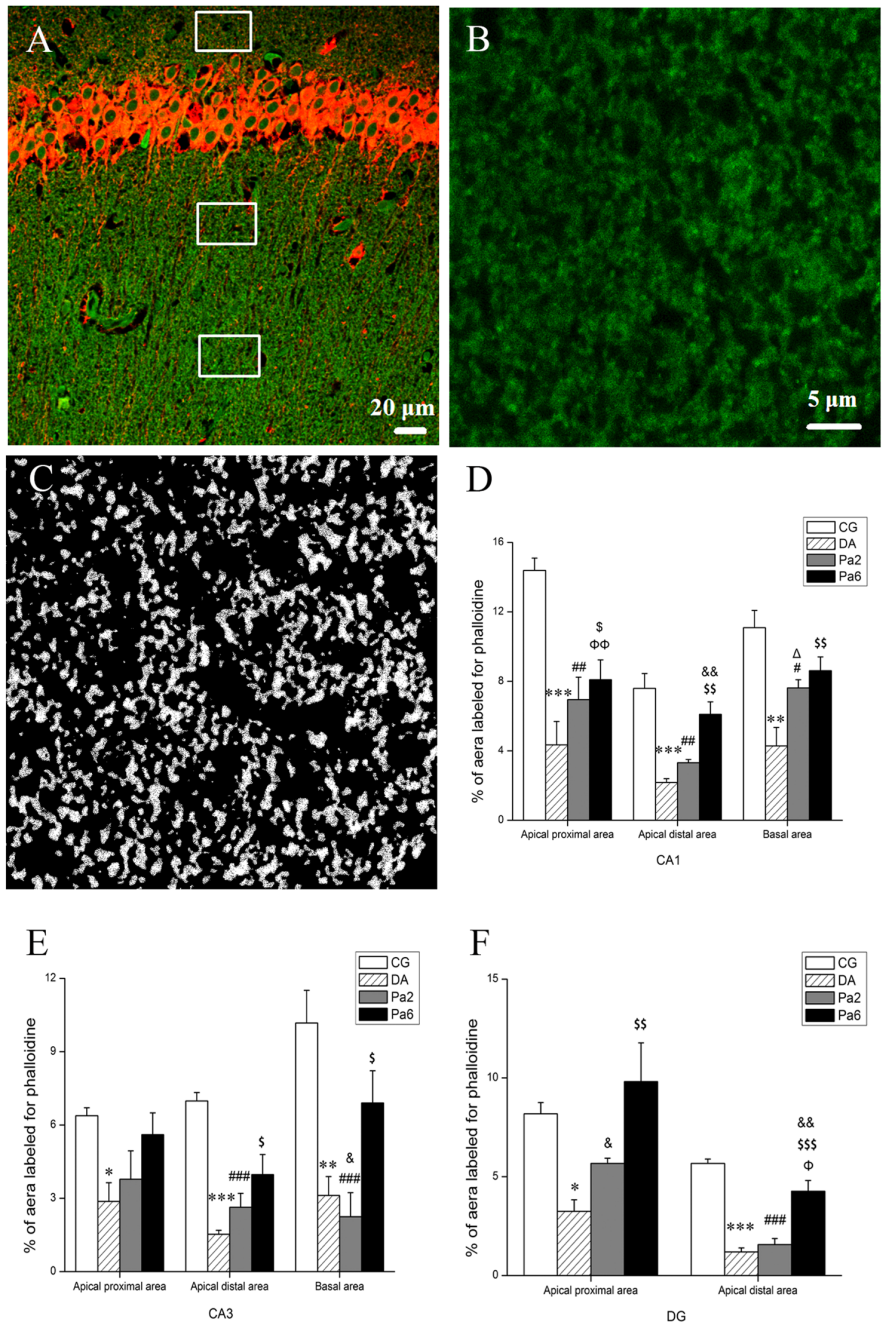
Effects of Pa on F-actin density assessed by phalloidin staining in different areas of hippocampus were analyzed with ImageJ. (**A**) A schematic diagram of the statistics area in the hippocampus. MAP2 immunoreactivity (red) was used to distinguish the different areas of the hippocampus. (**B**) Magnification of the statistics area in a single channel of 488 nm (OLYMPUS FV1000). (**C**) Image of (**B**) was processed using ImageJ software. The background was subtracted with a rolling value of 15, converted to 8-bit deep images and binarized using a determined threshold value (reduce noise 5, particles 2 - ~). The percentage of phalloidin immunopositive area in apical proximal and distal area and basal area in the hippocampal CA1 (**D**) and CA3 (**E**). (**F**) The percentage of phalloidin immunopositive area in apical proximal and distal area of DG. Data expressed as the means ± SEM (n = 3~5). Scale bar in (**A**) represents 20 μm and in (**B**) represents 5 μm. *P<0.05, **P<0.01, ***P<0.001, DA versus CG; ^#^*P<*0.05, ^##^*P<*0.01, ^###^*P<*0.001, Pa2 versus CG; ^Φ^*P<*0.05, ^ΦΦ^*P<*0.01, Pa6 versus CG; ^Δ^*P<*0.05, DA versus Pa2; ^$^*P<*0.05, ^$$^*P<*0.01, ^$$$^*P<*0.001, DA versus Pa6; ^&^*P<*0.05, ^&&^*P<*0.01, Pa2 versus Pa6.

The dendritic spine cytoskeleton is mainly composed of actin filaments at ~200 nm diameter [79]. Actin remodeling through actin filament nonequilibrium assembly and disassembly governs most, if not all, dendritic spine physiology [80]. Using phalloidin staining, we evaluated the F-actin density and determined that *D*-gal and AlCl_3_ induced reductions in F-actin in all statistical regions (**Fig 5**). Pa pretreatment for 6 weeks alleviated the decrease of F-actin in the apical distal dendritic region and the basal dendritic region of CA1 (**Fig 5D**), the apical distal dendritic region of CA3 (**Fig 5E**) and the apical distal dendritic region of DG (**Fig 5F**), whereas Pa treatment 2 weeks only alleviated the reduction of F-actin in the apical distal dendritic region and the basal dendritic region of CA1 (**Fig 5D**). The changes in F-actin in different regions of the hippocampus were similar to the alterations in the density of dendritic spines, which indicate that Pa alleviates *D*-gal and AlCl_3_-induced abnormalities of actin remodeling.

### Pa reduced p-cofilin1/cofilin1 ratio and relieved *D*-gal AlCl_3_-induced cofilin1 redistribution and rod-like formation

**Fig 6:**
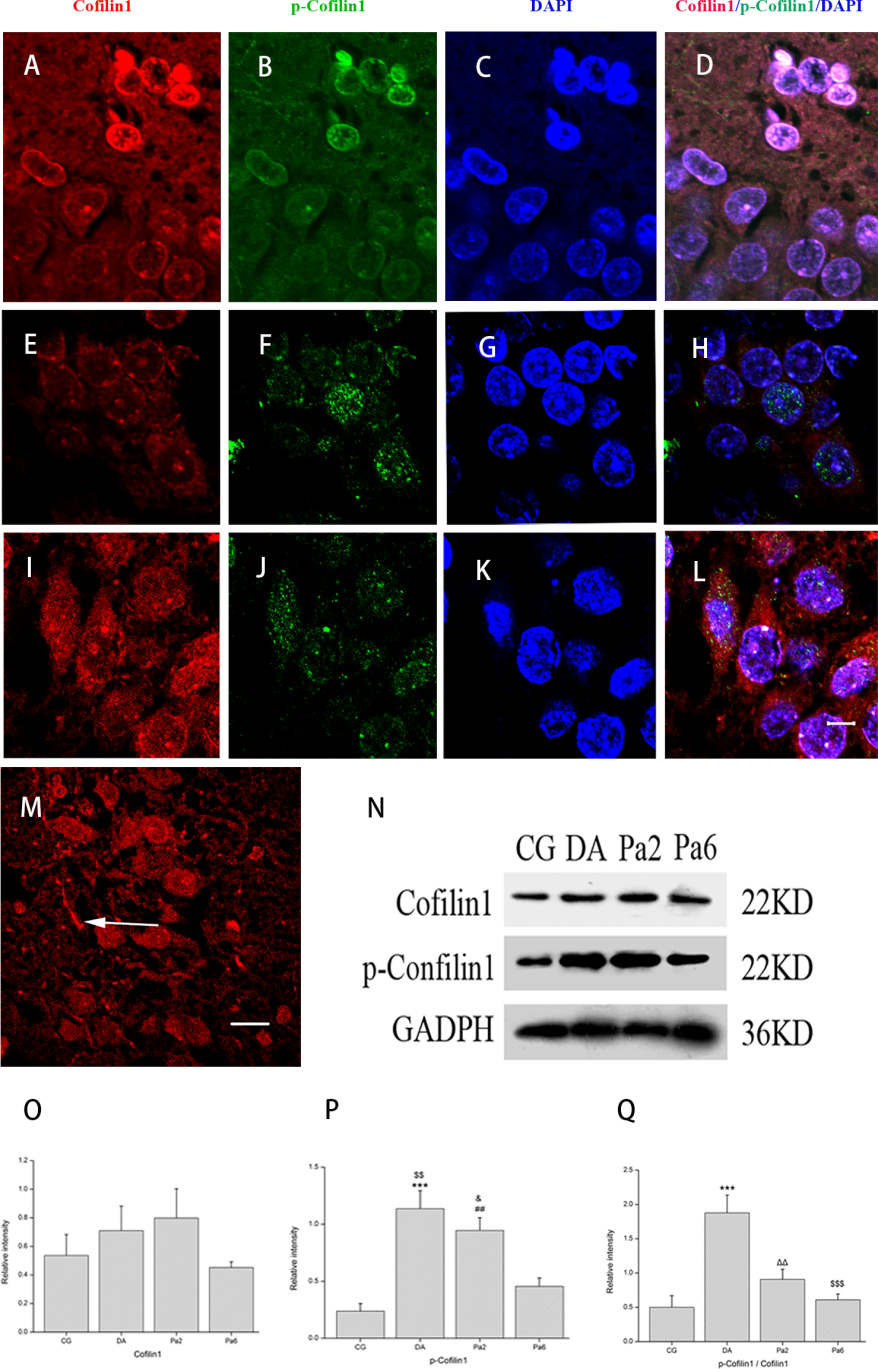
Effects of Pa on cofilin1- and p-cofilin1-immunoreactivity and their hippocampal levels. Immunofluorescence labels of cofilin1 (red, excitation wavelength 543/emission wavelength bp560-615), p-cofilin1 (green, excitation wavelength 488/emission wavelength bp500-530) and nucleus (blue, excitation wavelength 458/emission wavelength bp400-461) in DG of CG (**A-H**) and CA1 of DA (**I-M**). (**N**) Western blot for cofilin1 (sc-53934, USA) and p-cofilin1 on Ser 3 (bs-20261R, China) in CG, DA, Pa2, and Pa6. Pa downregulated the p-cofilin1 expression (**C**) and p-cofilin1/cofilin1 ratio (**D**); however, it did not affect the cofilin1 level (**B**). Arrows indicate rod-like inclusions. Data expressed as the means ± SEM (n = 3~5). ***P<0.001, DA versus CG; ^##^*P<*0.01, Pa2 versus CG; ^ΔΔ^*P<*0.01, DA versus Pa2; ^$$^*P<*0.01, ^$$$^*P<*0.001, DA versus Pa6; ^&^*P<*0.05 Pa2 versus Pa6. Scale bar represents 20 μm.

One of the best known regulators of actin remodeling is cofilin (mainly cofilin1) [81, 82], which may be inactivated by phosphorylation on Ser3 [83, 84] or release from membrane proteins [75]. Moderately activated cofilin is required for actin remodeling and synaptic plasticity; however, local excess activated cofilin may form actin-cofilin rods, which, in turn, lead to dendritic spine loss and dendrite atrophy [81, 85]. Aβ_1–42_ induces the formation of rods via activation (dephosphorylation) of cofilin in cultured hippocampal neurons [86]. Forebrain-specific deletion of cofilin leads to impairment of all types of associative learning [87]. Therefore, we investigated cofilin1 expression and distribution in the hippocampus. In this study, Western blot analysis showed that the p-cofilin1 (Ser 3) levels were significantly different between the groups (*P<*0.01), whereas the cofilin1 levels were not different between the groups (*P>*0.05); however, the administration of Pa for 6 or 2 weeks significantly lowered the p-cofilin1 levels and p-cofilin1/ cofilin1 ratio (p-cofilin1 levels: DA vs Pa6, *P<*0.01; p-cofilin1/ cofilin1 ratio: DA vs Pa2, *P<*0.01; DA vs Pa6, *P<*0.001) (**Fig 6N–6Q**).

Immunofluorescence technique indicated that cofilin1 immunoreactivity was predominately distributed in the cytoplasm- and nucleus- perimembranes and processes particularly in the cells morphologically similar to the neural stem cells (NSCs) in the subgranular zone (SGZ) of the dentate gyrus in the CG group (**Fig 6A and 6E**). *D*-gal and AlCl_3_ induced an increased distribution of cofilin1 in the cytoplasm (**Fig 6I**). Rod-like inclusions may be observed in neuronal processes (**Fig 6M**). Immunopositive p-cofilin1 primarily distributed in nuclei in the CG (**Fig 6B and 6F**), and *D*-gal and AlCl_3_ promoted the distribution of p-cofilin1 in the cytoplasm (**Fig 6J**). Pa pretreatment for six weeks relieved *D*-gal and AlCl_3_-induced cofilin1/p-cofolin1 redistribution and attenuated the rod-like formation. Co-immunoreactivity of cofilin1 and p-cofilin1 was detected in some nuclei or processes both in the CG and DA groups. These findings suggest that both cofilin1 activation by release from the membrane and cofilin1 inactivation by phosphorylation on Ser 3 may be involved in actin remodeling and neuronal damage processes.

### Pa downregulated RAC1/CDC42 expression in the hippocampus

Small GTPases of the Rho family, such as RAC1/CDC42, mediate the effects of Aβ on actin cytoskeleton dynamics by affecting cofilin activity [88, 89]. Therefore, we further examined the effects of Pa on small GTPases of the RHO family, including RAC1, CDC42 and RHOA.

**Fig 7:**
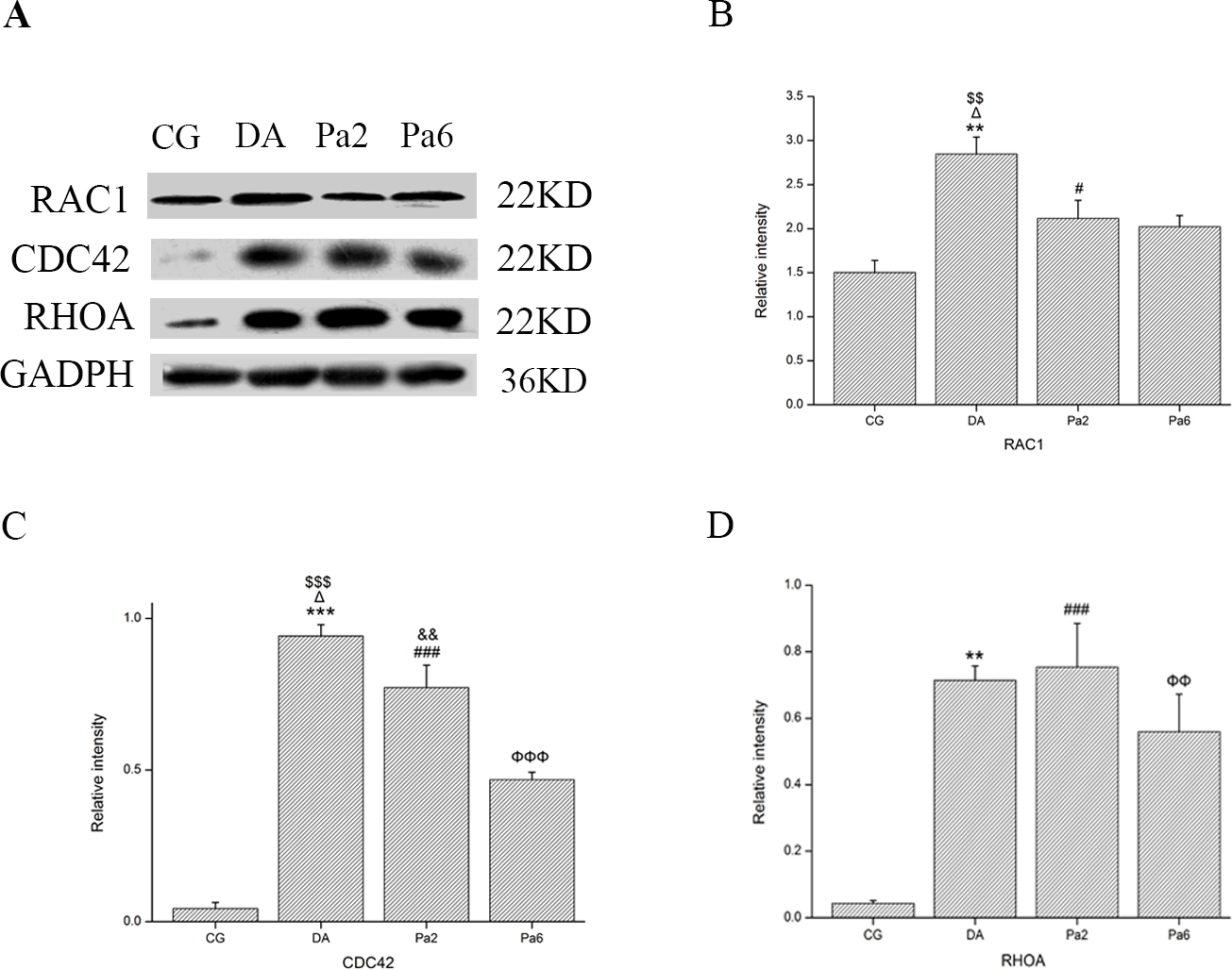
Effects of Pa on RAC1, CDC42 and RHOA. (**A**) Western blot bands of the hippocampal tissues determined with RAC1, CDC42, and RHOA antibodies in CG (left column), DA (middle left column), Pa2 (middle right column), and Pa6 (right column). GADPH (36KD) is an internal reference. Pre-treatments with Pa (50 mg/kg, i.p.) for 6 weeks significantly alleviated *D*-gal and AlCl_3_-induced upregulation of RAC1 (**B**) and CDC42 (**C**); however, it did not significantly reduce the expression of RHOA (**D**). Treatment with Pa for 2 weeks also significantly alleviated *D*-gal and AlCl_3_-induced increase of CDC42. Data expressed as the means ± SEM (n = 3~5). **P<0.01, ***P<0.001, DA versus CG; ^#^*P<*0.05, ^###^*P<*0.001, Pa2 versus CG; ^ΦΦ^*P<*0.01, ^ΦΦΦ^*P<*0.001, Pa6 versus CG; ^Δ^*P<*0.05, DA versus Pa2; ^$$^*P<*0.01, ^$$$^*P<*0.01, DA versus Pa6; ^&&^*P<*0.01, Pa2 versus Pa6.

Western blot analysis showed that the combined induction of *D*-gal and AlCl_3_ increased the expressions of RAC1 (**Fig 7B**), CDC42 (**Fig 7C**), and RHOA (**Fig 7D**). Pre-treatment with Pa for 6 weeks did not induce a significant reduction of RHOA (DA vs Pa6, *P*>0.05); however, it decreased RAC1 (DA vs Pa6, *P<*0.01) and CDC42 (DA vs Pa6, *P<*0.001) expression. Treatment with Pa for 2 weeks also significantly decreased RAC1 (DA vs Pa2, *P<*0.05) and CDC42 (DA vs Pa2, *P<*0.05) expression, which suggests that the effects of Pa on *D*-gal and AlCl_3_-induced disturbance in actin cytoskeleton dynamics may be mediated by RAC1/CDC42.

## Discussion

In AD patients, the major causes of cognitive impairments are considered to be neurite atrophy and synaptic loss, which are caused by toxic Aβ, particularly Aβ oligomers [70, 90–92]. In this study, the novelty of our work was to show that Pa alleviated *D*-gal and AlCl_3_-induced behavior damages as evaluated by the OFT (**Fig 1A–1C**), EPM (**Fig 1D–1F**), and MWM (**Fig 1G and 1H**). These behavioral disorders may be caused by *D*-gal and AlCl_3_–induced excessive productions of Aβ oligomers at ~28, ~36, and ~56 KDs (**Fig 2G–2I**) because these Aβ oligomers, particularly the ~56KD Aβ oligomer [13, 14, 70], highly correlate with impaired behaviors [93]. Consistent alterations with the Aβ oligomer changes included the dendritic spine density (**Fig 4**) and dendritic length and branching in our study (**Fig 3**). The alterations of dendritic spines highly concurred with the dynamics of the actin skeleton, accompanied by the disturbance of cofilin1 activity and the corresponding changes in RAC1/CDC42. These data suggest that Pa can deter formations of detrimental Aβ via the RAC1/CDC42 pathway, cause the redistribution of cofilin1 and decrease of the p-cofilin1/cofilin1 ratio (**Fig 6**), reduce rod-like formation (**Fig 6**) and synaptic and dendritic loss (**Figs 3 and 4**), and ultimately improve the behavioral defects.

Different forms of Aβ, including fibrillar amyloid β (fAβ) [94, 95] and soluble Aβ oligomers (sAβ) [19, 75, 96], have been reported as key players involved in AD pathogenesis [97]. They may cause dendritic spine loss and dendrite atrophy [17, 19, 98]. In rat hippocampal slices, acute overproduction of axonal or dendritic Aβ from APP/tomato-expressing viral-infected neurons reduces the spine density and plasticity at nearby dendrites [77]. Spine loss in number and shift of shape from mushroom to stubby are also observed in organotypic hippocampal slice cultures from Aβ of amyloid precursor protein transgenic mice [99]. Both in over-expressed human APP animal models and AD patients, a significant reduction in the number of dendritic spines and changes in dendritic morphology have been reported [17]. The present evidence by Golgi staining and three-dimensional reconstruction of the series of pictures also showed that with the increase of Aβ oligomers, significant decreases in the dendritic length and spine density were detected in the DA group, consistent with the previously described studies.

Pa treatment (50 mg/kg, i.p.) consistently increased the dendritic length and branching and the dendritic spine density in all assessed regions of the hippocampus (**Figs 3, 4B, 4F and 4H**); however, the effects of Pa on the proportion of different types of dendritic spines in different regions were different (**Fig 4C, 4E, 4G, 4I and 4J**), which suggests a selective role of Pa is mainly to attenuate the loss of mushroom/branched spines, the main postsynaptic type of functional mature spines.

Previous studies have indicated that the spine density of cultured hippocampal neurons is approximately 0.6/μm, the largest proportion of which is the stubby type spines, approximately 70% [100, 101]. However, electron microscopy studies have shown that the largest proportion of hippocampal CA1 spines in adult rats is the thin type spines, approximately 60% [102]. In this study, the spine density was approximately 1.2/μm, and the largest proportion was the mushroom / branched spines, approximately 50%, in the CG group (**Fig 4**). Morphological changes of dendritic spines, such as spine maturation, newborn, shrinkage, and devastation, are mainly dependent on the remodeling of the cytoskeleton protein actin [103–105], which is spatiotemporally regulated by numerous actin-binding proteins and upstream signaling molecules [106]. In mammal neurons, cofilin1, an actin-depolymerizing factor (ADF)/cofilin family protein, is widely considered to play an essential role in actin filament dynamics and reorganization [107]. Cofilin1 may be activated by the release of cofilin1 from PtdIns (4, 5) P2 or cortactin or the dephosphorylation of cofilin1 on Ser3 [75]. However, whether an excessive activation [28, 86, 108–111] or inactivation [87, 95, 112–116] of cofilin1 affects the actin-dependent synaptic plasticity and changes the spine shape remains quite controversial [117].

Our results showed that *D*-gal and AlCl_3_ did not affect the cofilin1 level; however, they promoted the release of cofilin1 from the plasma membrane into the cytoplasm and increased the formation of rods, which in vitro may be induced by excessive levels of active cofilin [109]; these factors were reversed by Pa pretreatment, which suggests that the excessive activation of cofilin1 may be only local particularly in some neuronal processes where rod-like inclusions can block intracellular transport and induce synaptic loss [116]. Different cell types have their own distinct mechanisms of cofilin regulation, and different compartments in one cell have uncoupled regulatory events of cofilin activity [75]. High-resolution imaging has indicated that increased levels of cofilin phosphorylation are a result of cofilin activation by dephosphorylation-independent mechanisms [118]. *D*-gal and AlCl_3_ upregulate the p-cofilin1 level and p-cofilin1/cofilin1 ratio and increase cytoplasmic distribution, which is consistent with the proposition that cofilin phosphorylation is involved in recycling cofilin back to the initial starting point in its activity cycle and spatially restricting cofilin activity [119–121]. Therefore, cofilin1 activation and inactivation may both be involved in actin-dependent synaptic plasticity, and Pa attenuation of the *D*-gal and AlCl_3_ induced-cofilin1 activation/inactivation is a dynamic spatio-temporal process, not a constant result [98].

It is well-established that extracellular signals, such as Aβ, regulate actin dynamics through the Rho GTPase family. Numerous studies have shown that Rho GTPase family members, RAC1 and RHOA, along with CDC42, work in a coordinated fashion to regulate cell functions, including the regulation of the actin cytoskeleton, cell polarity and migration, gene expression, and cell proliferation [122–126]. Rho GTPases, as molecular switches, bind downstream signals and initiate multiple signaling pathways [127]. Our results that Pa attenuated *D*-gal and AlCl_3_-induced upregulation of RAC1/CDC42 suggest that these molecules may be involved in Pa affecting the disturbance of Aβ on actin dynamics and support the hypothesis that cofilin-rod formation disrupts microtubule integrity, blocks intracellular transport, and induces synaptic loss and dendritic atrophy [128].

## Supporting information

S1 Fig Measurement and classification steps for dendritic spines. (DOC)
