# Supplementary material for: Novel derivative of Paeonol, Paeononlsilatie sodium, alleviates behavioral damage and hippocampal dendritic injury in Alzheimer's disease concurrent with cofilin1/phosphorylated-cofilin1 and RAC1/CDC42 alterations in rats
Source: PLoS One. 2017 Sep 21;12(9):e0185102. doi: 10.1371/journal.pone.0185102 (PMC5608314; doi:10.1371/journal.pone.0185102)
Supplement: S1 Fig — (DOC) [file pone.0185102.s001.doc]

**Supplement**

Golgi-cox staining images were analyzed by the reconstruction software and the spines were divided into three categories automatically. The specific steps are as follows:

1. Double-click the reconstruct
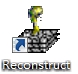
.

2. Series →newSeries.

3. Series →import images.


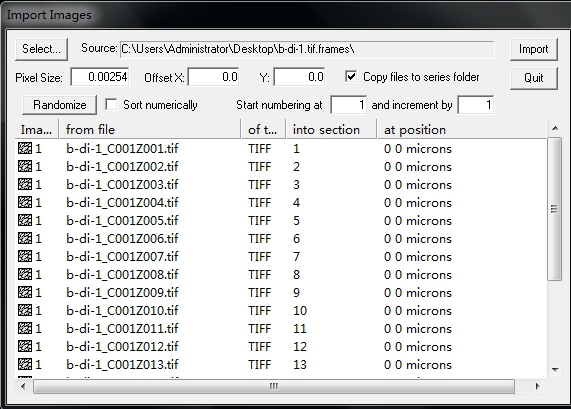


4. Main window in RECONSTRUCT. The purple rectangle indicates the dendritic segment chosen for analysis in this example.


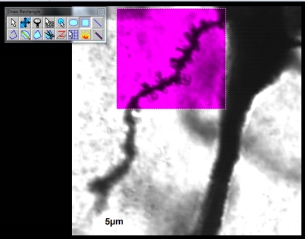


5. Zoomed in image of the chosen dendritic segment. The ‘Draw Line’ tool has been selected to create the straight length measurement.


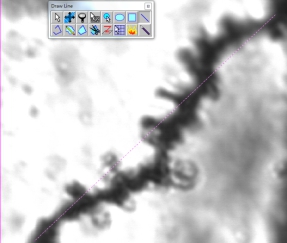


6. The ‘Draw Z-Trace’ tool must be used to measure the Z-length of the dendritic segment.


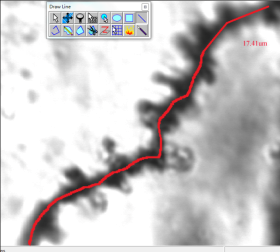


7. Draw a straight line across the width of the spine head and record how many spines have been measured.


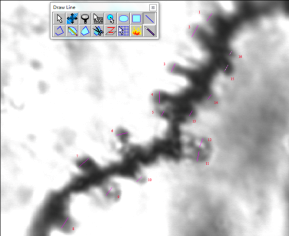


8. Drawing accurate Z-length measurements for spines often requires scrolling up and down through the Z-stack. In this example, the Z-trace starts at the base of the spine on Section a and terminates at the tip of the spine on Section b.


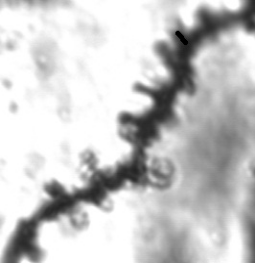

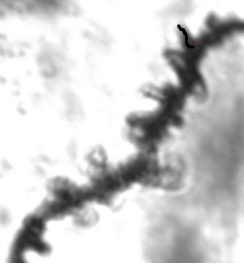


Section a Section b

9. Visualization of all Z-traces and straight line width traces for this segment.


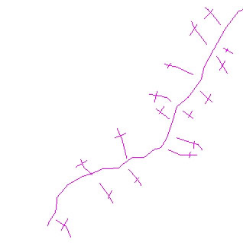


10. Constructing the data set: The syntax for the formula is as follows:

=IF(ISNUMBER(SEARCH(“branch”,E#)),”branch”, IF(G#>2,”filo”,IF(F#>0.6,”mush”, IF(G#w1,”long thin”,IF(H#>1,”thin”,”stub”)))))

Where # is the row number, E is the Trace Name, F is the spine width value, G is the spine length value, and H is the length:width ratio (LWR). The formula is hierarchical, classifying spines in the following order: 1) ‘‘branch’’ for branched spine, when ‘‘branch’’ appears in the trace name as entered manually by the user; 2) ‘‘filo’’ for filopodia, when the length value >2 μm; 3) ‘‘mush’’ for mushroom spine, when the width value >0.6μm; 4) ‘‘long_thin’’ for long thin spine, when the length value >1μm; 5) ‘‘thin’’ for thin spine, when the LWR value >1; 6) ‘‘stub’’ for stubby spine, when the LWR value ≤1.


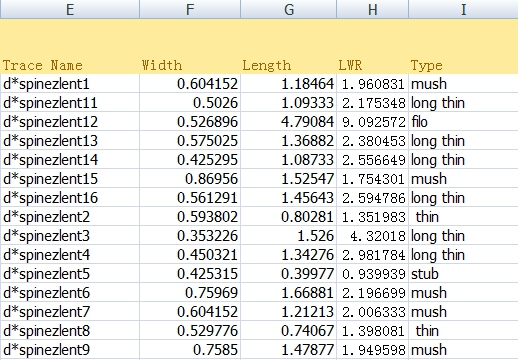


11. And then classified the results of these studies as three major categories so as to use statistics easily:1) Mushroom/ Branched,Width>0.6μm or “branch”; 2) Stubby，Length:Width Ratio<1，Length<1μm; 3) Filopdia/Thin，Length>1μm or LWR value >1.
